# The Outbreak of Smallpox among Sheep in Wiltshire

**Published:** 1862-10

**Authors:** 


### Art. VII.—THE OUTBREAK OF SMALLPOX AMONG SHEEP IN WILTSHIRE.

The circumstances attending the recent outbreak of smallpox among sheep in Wiltshire are of unusual interest. The facts, so far as at present known, are as follows:—

In July last, a fatal form of sickness broke out in the flocks of Mr. Joseph Parry, of Allington, near Devizes. At first, scattered instances of the malady occurred in a flock containing about 300 two-year-old ewes. In a few days, the sickness increasing, the lambs of this flock, which had up to this time been folded with their dams, were separated, put with others, and turned amongst the general breeding flock, making altogether above 1000 ewes and 700 lambs. In the course of a fortnight, the sickness showed itself also among this flock, affecting the lambs as well as the ewes. It is not quite certain from the accounts whether the time of outbreak in the general breeding flock is calculated from the first occurrence of sickness among the two-year-old ewes, or from the time of mixing the lambs of that flock with the larger flock. Presently the sickness increased to so great an extent, that "for days in succession as many as twenty and thirty of the ewes died in the most loathsome state of disease, their bodies covered with pustules, and a viscous matter running from the nose and from the eyes, rendering the sheep completely blind, and emitting the most foul stench."*

Alarmed with the rapid spread and malignancy of the disease; seeing that whatever he could do to check or alleviate it was of no avail; ignorant of its nature; and, according to the authority just cited, "finding that no one in the neighbourhood could give any satisfactory explanation as to the nature of the malady," Mr. Parry sought the aid of Professor Simonds, of the Royal Veterinary College. This gentleman, on the symptoms of the disease from which the sheep were suffering being detailed to him, at once concluded that the malady was *smallpox,* a conclusion fully confirmed on a subsequent inspection of the ailing flock. But when the Professor sought to trace the origin of the outbreak, he was foiled.

Hitherto it has been held of the smallpox of sheep as of the same disease in man, that it has never appeared (that is, within cognisable periods,) except there was some traceable or probable source of communication by contagion: that, indeed, the disease never arose spontaneously. In the present instance, however, the introduction of the malady into the affected flock seemed pretty nigh impossible, at least, highly improbable. No additions had been made to the flock from without, and no rams hired for two years. The disease could not have been imported by the shearers, as all the flocks they had worked among this year were known, and were ascertained to be and to have been free from the slightest symptom of the disease. No communication with the sheep of any strange flock, moreover, appeared to have or could well have taken place. But, most conclusive of all, it was not known that smallpox had occurred at all among the flocks of this country for several years.

Smallpox (*variola ovina*) is a disease of very recent date among sheep in England. It first broke out in the vicinity of London, in the autumn of 1847. In 1848-49 and 1850 the affection spread among the flocks of many counties, and caused immense losses. The origin of the malady, in this instance, could clearly be traced to the importation of several infected sheep from the continent, where it has long prevailed, more or less, and is never, perhaps, entirely absent from the wide district from which sheep are brought for our markets. The disease first declared itself in a small flock of sheep which had been imported, and sold to different purchasers in Smithfield Market. From

---

* *Devizes and Wiltshire Gazette*, quoted in the *Veterinarian* for September.

these sheep, and others imported about the same time, all doubtless coming from an infected district of the continent, and many of which were seized with smallpox within a fortnight after landing, the disease was derived, and spread to our own flocks. Notwithstanding, however, the formidable nature and wide extent of the epizoötic which followed, since 1853 (according to the writer of the article on "Sheep" in Morton's *Cyclopædia of Agriculture*), the affection has disappeared. Hence the recent outbreak of smallpox in the flocks at Allington would appear to be involved in mystery; and from the asserted improbability of its importation from a foreign source, and the absence of all evidence whatever of the existence of the malady as a sporadic affection among our own flocks, it would seem as if the conclusion were almost forced upon us, that the outbreak may have arisen spontaneously. "Hitherto," writes Professor Simonds,* "we have regarded smallpox as spreading only by contagion; now we must ask whether it can have a spontaneous origin? If the latter, no flock is safe, nor any part of the country secure. That which has occurred in Wiltshire may happen to-morrow in Sussex, and the next day in the Midland Counties. Having been consulted on the discovery of the disease by the owner of the animals, we have pretty well exhausted our stock of surmises for its introduction without making much progress towards a satisfactory explanation of the circumstance. The part of Wiltshire where the malady made its appearance is peculiar for its isolated position. No foreign sheep travel its roads, nor have any been known to be purchased by the butchers of the neighbourhood. The flock, consisting of 992 ewes, 9 rams, and 710 lambs, in which the disease originally manifested itself, has had no fresh animals, male or female, introduced into it, nor has it been commingled in any way with other persons' sheep."

The possibility of a spontaneous origin of the disease would also seem to be indicated by a subsequent outbreak in a flock at some little distance from Mr. Parry's farm. Up to August the 18th, the disease had been entirely confined to Mr. Parry's flocks; but on that day, a flock of 400 fat wethers belonging to a Mr. Harding, of Etchilhampton, showed signs of infection. The spot where the sheep were folded was about a mile and a half distant from Mr. Parry's farm, and in the intermediate space were other farms occupied by flocks belonging to different proprietors, and every care had been taken to prevent either direct or mediate communication with Mr. Parry's flocks: as an additional precaution, the sheep had been driven to the part of Mr. Harding's

* *Veterinarian*, September, 1862, p. 586.

farm most distant from Mr. Parry's. The flocks on the intermediate farms still, we believe, remain unaffected.

Since the irruption of the malady among Mr. Harding's sheep, the disease has appeared in other flocks in the same district, and also in Berkshire. But in the later instances of the outbreak, there is too good reason to believe that the disease was communicated to the healthier flocks by lambs or sheep purchased from flocks which were infected, but which up to the time of sale had not shown any indications of the disease.

Obscure as the first origin of this outbreak may at present appear a little consideration will, however, show, that the probability of the disease having been communicated, is by no means exhausted. The fact of the nature of the malady ravaging Mr. Parry's flocks not being recognised until Professor Simonds had been consulted, throws the gravest doubts on the assumption of the disappearance of smallpox among our flocks since 1853, or, at least, in more recent years. The first introduction and ravages of smallpox among the flocks of this kingdom must have fallen under the immediate observation of very many of our present sheep-farmers; the disease, moreover, is one of the most glaring character, and by no means difficult of diagnosis, even by the uninitiated. Hence it is far from improbable that sporadic smallpox may have occurred from time to time among our flocks, although not coming under the notice of a qualified veterinary practitioner.

Again, the possibility of infection from a foreign source is not entirely shut out, notwithstanding that since the outbreak of 1847-50 an inspection of all foreign sheep landed in England has been enforced. Although no instance of the disease has been reported from any of our ports, it is not to be forgotten that there are no means of ascertaining from what districts of the continent the sheep originally come, that is to say, whether from infected or healthy districts. Further, only such sheep are stopped as exhibit manifest tokens of ill-health. But, as in the first outbreak, the earliest indications of sickness may not be shown until a fortnight or three weeks after landing. Therefore, the practicability of the disease having been casually introduced in this fashion is not to be set aside without special inquiry.

It may be urged, however, that in either case the difficulty of tracing the outbreak in Mr. Parry's flock to communication would not be in the least lessened, from the peculiar circumstances and isolated conditions of the flocks. But a very serious doubt has been cast upon the completeness of this isolation by Principal Gamgee, of the New Veterinary College, Edinburgh. He avers,*

---

* *Times*, September 10.

from personal inquiry, that there is a free communication between different parts of the adjoining country and the Downs of Wiltshire. "There is," he states, "a drift along the celebrated Wansdyke which has been repeatedly the cause of the spread of contagious diseases from affected cattle and sheep traversing the Downs in order to avoid the payment of tolls on the high road. The public may rest assured (he adds) that there is no ground at all for the belief that smallpox could break out in Wiltshire spontaneously."

Apart from the doubts suggested by the details themselves, and Mr. Gamgee's statements, there are others arising out of the known or presumed phenomena of contagious diseases, which cannot be altogether ignored. The doctrines of the extreme school of contagionists (if we may so term the holders of these opinions) have been so admirably summed up in the columns of a contemporary,* in reference to this outbreak of smallpox, that we cannot do better than cite them as there expressed. Writing of the theory of spontaneous origin of the outbreak, it is said—

"Is that possible or feasible? In the presence of all the known facts respecting contagious diseases, we must express our entire disbelief in the theory of spontaneity. The same law extends to contagious diseases as is afforded in the propagation of animal life; *i.e.*, there can be no new presence without an antecedent, or, in plainer words, something cannot arise out of nothing. The first of these sheep attacked, at some particular moment, was a healthy animal; it became diseased, and as a result of that disease yielded products poisonous in character, which produced the same changes and the same products in other sheep: *ergo*, the first sheep must either have been subjected to a similar poison, or the others did not suffer from the contagion of the first. To admit any difference in this argument is to assume that two causes can produce one phenomenon, which is absurd. The spontaneous origin of the disease is, therefore, the last hypothesis that can be accepted; before it there are two others, either of which, however improbable, have at least the merit of not being impossible. The first of these is to the effect that a germ of the disorder was conveyed to the animal first affected, a germ derived from some other animal previously affected. How the germ was carried it would be speculative to suggest, but the possibilities of conveyance are within the range of experience. These poisons, indestructible at common temperatures, and solid in their form, have a susceptibility of adherence to other solid bodies, and may be intentionally or accidentally carried, retaining their poisonous properties for any number of years, over the whole universe; consequently, although the probabilities were as a million to one against such conveyance, the possibilities remain."

The writer then proceeds ingeniously to suggest, the possible relationship of the disease which had broken out among Mr. Parry's sheep to Farcy. We presume, however, that now any

* *Social Science Review*, August 16, 1862.

doubt as to the actual nature of the affection, and as to its being *variola ovina*, is fully set at rest.

A logical deduction holds good only so far as the premises upon which it is based are sound. Now, the assumption that the propagation of animal life and of contagious diseases is governed by the same law, is open to serious question. The analogy is no doubt most plausible; but this is not sufficient to justify the assumption. Recent experiments have again conclusively shown (a doubt having once more arisen on the subject,) that animal life is never known to be generated without a presumption amounting almost to absolute demonstration of a pre-existing germ. But we are not aware that any like proof, or equivalent probability, has been shown of the pre-existence of germs of at least two diseases, one of which, under certain conditions, is highly infectious, and the other is both contagious and infectious in the utmost degree—we allude to typhus and puerperal fever. These instances alone serve to vitiate the analogy and its logical consequences. Probability, not possibility, is the rule of analogical argument in physical science; and the possibility of the laws of one series of phenomena being similar to the laws of another and very different series, (so far as at present known,) cannot be admitted without the extremest caution, and then only as a guide to research, not a limitation. At every step we are taught caution in the use of analogical argument in pathology. The purulent ophthalmia of infants may unquestionably be occasioned in parturition, and there is no doubt that once developed in a single individual, it may be widely propagated to others by the direct application of the purulent discharge, and probably also by diffusion of particles of this discharge through the atmosphere. It can scarcely be admitted that the causes of the first and subsequent cases are identical. The same is true of acquired and propagated purulent ophthalmia in man, and of certain forms of gonorrhœal discharge.

It is undoubtedly correct that we do not know the limit of retention of deleterious properties by contagious or infectious matters. In smallpox and scarlet fever, for example, particularly the latter, we know that the poisonous emanations from the sick maintain their active properties for long periods. But we must not confound the question of limitation with that of indestructibility. It is to be remembered that the contagious virus diffused from the sick has never yet been separated from an infected atmosphere, and subjected to scientific analysis. Much may be inferred from its effects; but much also lies hid. If we permit ourselves, then, to adventure conclusions other than those which are warranted by the facts actually within our grasp, we run the risk of endless confusion.

The occasionally observed prolonged retention of virulent properties by the poisonous emanations or secretions of certain specific diseases must, in seeking the conditions determining any given outbreak of such diseases, not be lost sight of, and must, among other considerations, influence our inquiries. For example, the poisonous emanations or secretions from epizoötic pleuro-pneumonia or eczema, will long infect stables, cattle-sheds, trucks, and even plots of ground. But it is manifest that to extend the conclusions from this fact beyond what is actually warranted by observation, can aid us little. For where we must, in the absence of all other evidence, fall back upon the doctrine of the "indestructiblity" of specific poison-germs for the explanation of any given outbreak, it is tolerably certain that the immediate conditions of the outbreak are so much more important than the theoretical, that we may well afford to let the latter rest in abeyance. The doctrine of "spontaneity" may never, perhaps, extend beyond this point, that, under certain conditions, all evidence of specific origin to a disease is lacking; but if the doctrine be made to imply more than this, like that of "indestructibility," it carries us into a region of speculation where ingenuity must supply the place of more sober research.

When, moreover, a contagious element is manifestly present, we err egregiously if to this we attribute the sole part in the propagation of disease. No word, in the whole range of medical science—human or veterinary—has done, or still does, more mischief than the word "contagion." Given *contagion*, and it is too commonly supposed, both professionally and popularly, that in this single fact we have all that is required to account for the propagation of a communicable disease. It cannot be too strongly insisted upon that contagion is not an absolute but a relative fact; that the contagious element is inoperative except under certain conditions. Remove the conditions, and the poison germ is powerless. No specific communicable affection is exempted from this law, not even smallpox or scarlet fever, although these diseases are least manifestly subjected to it. It is not sufficient therefore to determine, in investigating the extension of a communicable disease, that it possesses contagious or infectious properties, but also to determine under what conditions those properties come into play. Now it is in researches conducted to this latter end, that all the great discoveries and improvements in the control and prevention of epidemics have taken place, and which still promise the richest results to the investigator. Until a very recent period, it has been customary to lay the greatest stress upon, and to devote the most attention to, the exclusion of the poison germ. Even now, largely in medical, and more especially in veterinary science, the chief aim is concentrated upon measures to prevent the introduction of the contagious element. There can be no doubt that if this could be effected, the greatest conceivable protection from a disease would be obtained. But the practicability and efficiency of such a course must be learned from experience. What, then, does experience teach us on this subject? It teaches us that the strictest quarantine, and the most perfect sanitary cordon, have not prevented either the introduction, or spread, or development of any one of the great epidemic or contagious diseases in a State or country; that whenever, over large districts or in isolated localities, the attention has been centred solely upon the prevention of the introduction of contagious disease from without, that prevention has, as a rule, failed; that the most virulently contagious diseases have broken out within the limits of, and in spite of the most stringent protective observation, while contiguous districts and populations, entirely unprotected, have either escaped or suffered but very slightly. On the other hand, experience has also taught us that the introduction of a contagious disease into a given locality, under certain conditions, has led to a formidable extension of the disease; that the introduction of a like disease in another locality, under different circumstances, or into the previous locality at a different period, has been followed by no evil consequences, the disease dying out in the individuals primarily affected; and that the most efficient means of protection from a contagious affection, even when pestilential, has been the removal of the conditions fostering the development of the disease, not the attempted exclusion of the disease itself.

The conclusion is obvious, that any system which may be adopted for the prevention of the extension of a contagious disease will in all probability entirely fail, unless it provides not only for the exclusion of the disease, but also for the removal of the whole of the conditions upon which the further development of the malady depends.

That the latter is by far the most important point to attend to, a little reflection will show, even if the vast experience of many people and many epidemics and epizoötics does not alone suffice to convince. Consider for a moment the mode of manifestation of all great epidemics. A few scattered cases of the disease at an interval of, perhaps, several months; a few isolated instances of small but significant outbreaks—these are the usual forerunners of the great outbreak, and are commonly neither heeded nor even known. But to make a provision for the exclusion of the possibility of contagion during this period, would be permanently to interpose so serious an impediment to commerce and intercourse, as to make both the one and the other well nigh impracticable; and this for an end the attainmentof which is extremely doubtful, if not altogether improbable. Let this consideration be applied to efforts intended to prevent the introduction of disease from without, into the boundaries of a kingdom. And now of contagious disease within the limits. When diphtheria first attracted notice in this country, during the recent epidemic, the then known times and places of appearances of the disease, gave rise to the belief that it had first broken out in the south-eastern counties, and from them gradually spread by contagion over the remainder of the kingdom. The outbreak was, moreover, thought to be connected with a formidable irruption of the disease which had previously occurred in Boulogne. It was a noteworthy fact, that one of the earliest cases recorded on the south-eastern coast, was in a household which had but recently crossed the channel from the infected French city. Here then the fact of diffusion by contagion seemed most probable, if not clear. It is now, however, known that several scattered cases of diphtheria had occurred in various inland districts of England in 1848 and 1849; that in the winter of 1849-50, the disease prevailed to a most serious and fatal extent in Haverfordwest in Pembrokeshire; and that at least three other local outbreaks at different intervals of time preceded the general outbreak.* Again, and touching more closely on our present subject, notwithstanding the stringent sanitary police exercised in France over domestic animals suffering from contagious diseases, this has neither prevented the extension at different periods, nor terminated the ravages, nor saved any of the great sheep districts of France, from the ravages of smallpox. This disease has now prevailed as an epizoötic, now as an enzoötic; now it has infected the flocks of one district, now of another; now it has broken out in a comparatively mild, now in a malignant form, in spite of the best conceived and most ably governed sanitary police measures. Not until the disease has become painfully manifest, indeed, could a sanitary police be of any avail, and then chiefly in restraining somewhat the sale of plainly diseased animals, or of the animals from infected flocks, or of their flesh as food. Indeed, if it be difficult to exercise a strict and trustworthy restrictive observation over disease in man, how much more so will this be the case over numerous and vast flocks governed, too commonly, by ignorant herdsmen?

Now we would not have it thought that we conclude, therefore, that the restrictive efforts of a sanitary police in the prevention of epidemic or epizoötic disease are of little utility. Far from it. It is of the greatest importance, for example, to prevent the sale or distribution from one district to another of diseased animals, or diseased

---

* *See* Mr. Radcliffe, "On the Recent Epidemic of Diphtheria."—*Lancet*, July 12, 1862, p. 33.

flesh; this is unquestionable. But the argument is, that this alone is not to be depended upon; it is, indeed, utterly untrustworthy for the prevention of the spread of disease, however contagious, and for the protection of a healthy district. The same remarks hold true, in a great degree, of the internal police of flocks and herds when affected by a contagious disease. While separation and isolation of the affected animals may be pursued with great success, in restraining the propagation of the disease in small flocks favourably situated for the experiment; in large flocks, so numerous as to render the daily inspection of the individual animals a matter of almost impossibility, or which are kept on lands which do not permit of satisfactory segregation and isolation, these measures have proved of little or no avail.

It follows, therefore, that while giving just attention to measures of sanitary restriction, our chief hope in the restraint of any contagious epidemic or epizoötic must rest in ascertaining and controlling the conditions which are requisite for the development of its contagious properties. In several of the most fatal epidemics and epizoötics, as, for example, in the typhus or typhoid of man, and the so-called "contagious typhus" and "pleuro-pneumonia" of beasts, we know that the conditions for their extension by contagion depend upon certain well-known deficiencies in the sanitary circumstances under which the individual is placed, and which being removed, the influence of contagion or infection or both is rendered nugatory. It does not follow that these deficiencies are at all times capable of being readily removed, as in the case of an old-built, imperfectly-drained town, and ill-ventilated, badly sewered houses, fostering typhoid fever; or in the conditions of the vast herds of Western Europe or of Australia—the former liable to frightful murrains of contagious typhus, the latter recently decimated by epizoötic pleuro-pneumonia. The proposition is that these conditions are much more certainly under our control than the contagious germs of diseases, as shown by experience. On the other hand, we know that the conditions of development of other contagious diseases, as smallpox and scarlatina, are less dependent upon local circumstances than upon the state of the individual at the time. What that state may be we are as yet entirely ignorant of in both cases named, and in the latter case we are unhappily compelled to depend for protection upon isolation of the disease alone; and how sadly inefficient this protection is, and almost necessarily so, we need not say. In the former case we should be in a like unfortunate position, were it not that science had discovered that the disease may be induced in a modified form, and the predisposition to it, that is condition of development, thus exhausted.

What, then, should be the course of action to be adopted in the present outbreak in Wiltshire for the prevention, as far as practicable, or restraint of the further spread of the disease, or obviation of its chief evils ? Of the police measures prohibiting the sale of sheep within the infected districts, unless under the permission of a competent judge, the disinfection and purification of places occupied, temporarily or not, by affected animals, and also of the sale of diseased meat as a safeguard to the public, there can be no question; and an Order in Council (somewhat late in the day, it must be confessed) has authorized the local authorities to take all needful steps in carrying out such measures. Of the means to be adopted for the more immediate treatment of the diseased flocks little doubt can also be entertained, if what we have advanced be correct. There is, indeed, but one which offers absolute safety to the flock, or which gives a sure promise of mitigating the loss and anxiety of the farmer, to wit, Inoculation. But the practice of inoculation is liable to be attended by evils of so grave a character that it is not to be adopted without serious consideration. It is not, indeed, to be wondered at that at the first outbreak, a difference of opinion should have been manifested upon the propriety of its adoption by the chief authorities consulted on the question, Professors Simonds and Gamgee.

The question is one of immense moment both to the farmer and the public. It very seriously involves the substance of the farmer, and a staple article of food of the latter, as well as certain important manufactures. The value of the flocks in this country is estimated at no less than 100,000,000*l*.* But the loss to the farmer is not to be estimated solely by the numerical diminution of his flocks from the mortality occasioned by the disease. In this loss must be estimated the losses occasioned by the death of sheep fat and fitted for the butcher, of sheep selected and set apart for the improvement of breed, the dropping of lambs, the loss of lambs deprived of their dams, from the difficulties experienced in rearing them, the deterioration in the flock which recover the sickness and the impossibility of fattening them for a season, the loss of the wool, one of the most important losses, and finally, the occasional destruction of the eyesight of the animal. Add the expenses occasioned by the most ordinary care and attendance required during an outbreak of the disease, and some conception may perhaps be formed of the loss a sheep-farmer may be subjected to whose flocks have the misfortune to be attacked with smallpox.

The mortality among sheep affected by epizoötic smallpox ranges from 12 per cent. in the milder, to 50 per cent. in the graver outbreaks among different flocks. Occasionally the mortality

---

* *Times*, Letter from a Correspondent.

reaches even a higher rate, as much, indeed, as 60 or 70 per cent., as was observed in several flocks in this country in the outbreak of 1847-50. Generally an outbreak of epizoötic smallpox involves a loss of at least one-fifth of a flock. When the disease has become established among the flocks of a country, and is an annual visitant, or recurs at frequent intervals, the mortality may not exceed 7 per cent.

The mortality is affected both by atmospheric conditions and the state of the animal at the time of attack. It is found to be augmented either by great heat or by cold, by a low, wet or damp, and generally unhealthy location, by tender age, and by pregnancy. The animal being fatted, or in poor condition, or of a foreign breed, also exercises an unfavourable influence over the result.

It is most important to note, also, the usual mode of outbreak and progression of epizoötic smallpox among a flock of sheep. The disease does not attack one or two members of a flock, and from these spread gradually through the whole flock; neither does it attack a whole flock at once. It shows itself, like most epidemic diseases in man, in two, or three, or more successive attacks, each involving a previously unaffected and greater portion of the flock, and each extending over from twenty-five to thirty days, until the entire flock has been affected. Hence it follows that the duration of an outbreak of smallpox in a large flock commonly extends over three or four months.

These facts being premised, let us briefly consider the measures to be adopted for preventing the spread, or mitigating the ravages and losses arising from the disease.

Some of these require no comment. The prohibition of the vending, or exposing for sale, of sheep in a diseased state, is an initial step, as we have already remarked, admitting of no question. The same may be said of the destruction and careful burial of sheep hopelessly sick. These and all other measures imply the most thorough daily inspection of a flock practicable. There remains segregation of affected individuals and isolation of infected flocks, and inoculation.

The circumstances under which separation alone, whether of individual sheep or of infected flocks, or of both, are likely to prove available, may perhaps be best gathered from the following extract from one of several articles by Professor Delafond—translations of which were published in the twenty-first volume of the "Veterinarian." The articles are devoted to an examination of the question of inoculation of flocks of sheep for smallpox, as a measure of sanitary police. The opinions expressed by the learned professor, and the reasonings upon which they are based, deserve the highest consideration, and we would commend them to the attention of our readers for their scientific interest, in addition to their great practical importance at the present moment.

"So long," he says, "as the pox is confined to one flock, or in case even it should prevail among several flocks in the same commune or locality, isolation, whether it be in the pens or in the fields, is generally the only measure that can prevent the spreading of the malady. But when an epizoötic and fatal pox is spread over a large surface of country, during a warm and dry season, and at a time that the flocks are out grazing, and especially where natural obstacles, such as woods, hills, mountains, marshes, and broad streams of water, become no boundaries to the infected localities, folding and sequestration being difficult of execution, incessant and often impracticable surveillance are the only measures, and they sometimes fail in arresting the progress of contagion. The persistence of sheep-pox in 1812, in the department of the Somme, together with its introduction into the Pas-de-Calais, and its propagation in fifty-nine parishes (*communes*), the appearance of the malady in seventy-four parishes of the Marne, and its duration nine years in the district; its extension and continuance for two years, from 1820 to 1822, in the department of Aube and Herault, are, among other proofs which I could cite here, demonstrative examples that the sanitary measures prescribed according to the Acts of Parliament and regulations in force at the present day, particularly those of sequestration and folding, are insufficient in a great number of instances to arrest or confine the propagation of epizoötic smallpox."

These opinions, amply supported by other continental writers, very fairly indicate the circumstances under which sequestration may be expected to be of avail; at the same time they show under what circumstances its failure may be anticipated. It may be presumed from the opinions of Mr. Gamgee, as recorded in the daily papers, that the infected flocks in the vicinity of Devizes are so situated as to render segregation and isolation practicable and, to some extent, successful measures. On the other hand, the general character of the outbreak, and the malignancy of its first irruption in Mr. Parry's flocks, give a high degree of probability to the assumption that the disease is epizoötic in nature, and that this outbreak is but the forerunner of a more wide-spread manifestation of the disease. If this be the case, knowing the usual course of epizoötic smallpox among a flock, the gravest doubts may be entertained of the sufficiency of separation and isolation to save the flock in every instance, or to restrain the extension of the disease.

Separation and isolation failing, inoculation is our only resource. But before noting the value of this remedy, let us first see in what manner, and under what circumstances, English veterinary surgeons advocate its use. We quote here from that standard work, Stephens's "Book of the Farm," which, we believe, pretty accurately expresses the opinions most commonly held on this subject both at home and abroad:—

"We decidedly object to the plan of inoculation simply as a means of precaution, whilst a flock is free from the disease, as by this means we propagate an infectious disorder, though in a mild form. The plan we advise, after some experience and considerable reflection, is, as soon as the disease appears in a flock, to practise separation and examination as rigidly as possible, but at the same time to inoculate one or two sheep. Then, if we find that the disease extends in spite of our daily examination, we shall have from these inoculated cases favourable lymph for the inoculation of the remainder. If some twelve or twenty cases of smallpox have really occurred, then without any further delay we advise inoculation to be practised on the remainder of the flock; for it should be borne in mind that the earlier cases are generally mild, and the disease increases enormously in virulence and fatality as it extends. The advantages in favour of this plan appear to be these: we may select the most favourable weather for the operation, and in the course of six weeks are free from further anxiety about the matter; the utmost care can be taken of the flock during the period, and the greatest vigilance exercised to prevent the spread of the disease to other flocks—care and vigilance which it may be difficult to adopt through so long a period as the system of continual turning might demand. Besides which, it should be remembered that there are at least three ewes probably to one wether sheep, and these ewes being kept for breeding, it is of the utmost importance to select the earliest and most favourable time for receiving the disease, and not to run the risk of their getting the disease naturally just previous to lambing. It is quite a mistake to suppose that the risk of spreading infection is increased by inoculation; in fact, it is lessened, for the disease becomes milder, having a mortality ranging from two to ten per cent. It is also circumscribed, and necessarily entails the utmost vigilance, and prevents the sale of sheep from the flock for a given period—of twenty-one days."

The course here so clearly and, in our opinion, convincingly advocated, would appear to be substantially the same as that adopted by Professor Simonds in Wiltshire.

The advantages to be derived from the practice of inoculation under the circumstances recommended, are so well set forth in the extract we have just quoted from the "Book of the Farm," that it seems almost superfluous to reiterate them. It may be as well, however, to examine a little more specifically the objections advanced against the practice. The objection most persistently urged at the present moment is, that by inoculation the disease would be largely multiplied, and the chances of its spread and naturalization among our flocks increased. The answer to this is, that the measure is not to be had recourse to, neither is it advised, except when the disease has actually broken out among a flock, when separation and isolation have failed, and when there is a high probability of the malady running through the whole of the sheep. That, as a consequence, it substitutes a milder form of the disease for the natural disease, and limits the duration to a brief period of three or four weeks, obviating a probable duration of three or four months. In both ways, by the mitigation of the severity of the disease, and the limitation of its prevalence, it diminishes the risk of spreading—a risk still further diminished by the more complete and thorough care which can be exercised over an entire inoculated flock, than over a flock passing through the natural disease, and by the almost impossibility of an inoculated sheep being passed into the market, or otherwise disposed of.

It is also urged that inoculation proves disadvantageous by giving the disease to animals which might otherwise escape; by communicating probably as intractable and fatal a disorder as natural pox; and by consequently exercising as serious an influence as the natural disease on breeding, fattening, feeding, and the fleeces.

Inoculation is at the best a choice between two evils. But experience has shown that communicated smallpox, except under extraordinary and most rare instances of carelessness in inoculation, is more tractable in every way, and much less fatal than the natural disease. The numerous statistics of inoculation practised under every circumstance of age, condition, and season, collected by Professor Delafond, and confirmed by his own wide observation, show a mortality not exceeding 0·5 per cent.—1 in every 200 successfully inoculated animals! Further, his statistics also show that in no instance recorded has inoculated smallpox given rise to a disease, even under the most unfavourable circumstances of operation and care, as fatal as natural epizoötic small-pox. Again, the inoculated disease, from its mitigated form, gives rise to still other advantages. It limits the period when the fattening, breeding, and sale of the flock cannot be properly carried out, and dimishes the injurious effect upon the fleece.

In fact, inoculation substitutes a milder, more managable, less fatal, and less injurious disease, for the more formidable disorder already present, limits the duration of the infection among a flock within the least practicable period, and diminishes the probability of extension of the malady.

The adoption of inoculation seems, therefore, to be a remedy admirably fitted to control an outbreak, such as that which would appear to have occurred in Wiltshire, provided that the flocks operated on are so situated as to admit of satisfactory isolation. Under such a condition, the remedy promises the greatest safeguard from loss, both to the farmer and the public.

This is no question, we reiterate, of introducing a disease among hitherto healthy flocks, but of obviating, as far as practicable, the ill effects of a formidable affection already manifested among infected flocks, and when separation and isolation have been already tried and failed, or observed to be useless from the disease having become manifest in many sheep at the same time.

The results of inoculation on the Continent have proved so favourable, and the practice is thought to have exercised so salutary an influence in obviating and controlling epizoötic outbreaks of disease, that Professor Delafond has urged the importance of making its performance compulsory. Not the least of its benefits, it is asserted, have been the limitation and diminution of anxiety and immediate expense, as well as of ultimate loss to the farmer.

We are fortunate in possessing an able monograph on *variola ovina*, containing an account of the first introduction of the disease into England, by Professor Simonds;* and we would refer such of our readers as are desirous to become fully acquainted with its character and progress, to this valuable book. We are glad to have to state also that Professor Simonds has been instructed by Government to investigate and report upon the origin and progress of the present outbreak of the malady. Principal Gamgee is also pursuing an inquiry on his own behalf in the infected district. We shall look forward therefore with considerable confidence to a solution of the doubts we have expressed at the commencement of this article, and a complete history of this extraordinary outbreak, from the researches of these accomplished veterinary scholars and practitioners.

### POSTSCRIPT.

The preceding observations were already in the press, when we received more accurate information of the condition of the infected flocks, and probable sources of the outbreak in Wiltshire. It would appear, that Mr. Parry's sheep-walk and neighbouring walks are traversed by so-called " drifts," or bye-roads, frequently used by strange flocks and herds, to avoid certain tolls on the highway; that from the passage of strange flocks scab has been communicated to healthy flocks feeding on the sheep-walks traversed, and from the passage of infected herds, vesicular murrain to herds feeding on the Downs; that the introduction of smallpox from a foreign source is therefore highly probable. Moreover, the manifestation of the disease hitherto,

* *A Practical Treatise on Variola Ovina, or Smallpox in Sheep.* Churchill, 1848.

among the flocks in the neighbourhood of Allington, may be accounted for without the assumption of any unusual or epizoötic sensitiveness of the sheep to the malady; and it is most probable that, had the earliest cases among Mr. Parry's flock been recognised, and the animals immediately killed, the extension of the disease would have been stayed. It would seem, also, to be highly probable, that the further course of the disease among a flock, when it has once shown itself, may be at once stayed or held in thorough check, by active and complete professional inspection, and the separation and destruction of the affected animals, without having recourse to inoculation. It further appears, that inoculated flocks, through carelessness or inadvertency of the shepherds, have communicated the disease to other flocks; and that from the crowded state of the Downs, due isolation of an inoculated flock is very difficult, and in certain cases, perhaps, impossible.

From these facts it would seem, then, that inspection and separation should be chiefly depended upon in the present outbreak, and inoculation only had recourse to as a final resort. It would also seem that if the outbreak should still extend, it would become a question for consideration whether or not inoculation should not be subjected to legislative interference,—not for the purpose of prohibiting the measure, as some have advised, but of regulating its uses. For it would be requisite to forbid the performance of the operation by an unqualified person, and to compel, under heavy fines, the proprietors of inoculated flocks to entirely isolate their flocks. It is asserted that one or more flocks have been inoculated in the present outbreak, in which no indications of smallpox have appeared. We shall be curious to learn what could have induced this course to have been taken.

If there is no legal right of way for strange flocks and herds along the "drifts" traversing the Downs, ought not the farmers to take some measures to prevent these bye-paths being made use of by any but those who have a right of passage? Or, if a general right of way exists, should not steps be taken to prevent or diminish the probability of diseased sheep or cattle travelling along these roads, which are fully open to the grazing tracts?

